# MicroRNA array and microarray evaluation of endometrial receptivity in patients with high serum progesterone levels on the day of hCG administration

**DOI:** 10.1186/1477-7827-9-29

**Published:** 2011-03-06

**Authors:** Rong Li, Jie Qiao, Lina Wang, Li Li, Xiumei Zhen, Ping Liu, Xiaoying Zheng

**Affiliations:** 1 Peking University Third Hospital, No. 49, North Huayuan Road, Haidian District, Beijing 100191, PR China; 2 Institute of Population Research, Peking University, No.5 Yiheyuan Road Haidian District, Beijing 100871, PR China

## Abstract

**Background:**

To determine the effect of higher progesterone (P) level on endometrial receptivity.

**Methods:**

This was a prospective analysis conducted in the Reproductive Medical Center of Peking University Third Hospital. All patients received IVF treatment and canceled embryo transfer in the same cycle and were divided into group 1 (normal P; 7 patients) and group 2 (elevated P; 12 patients). Endometrial biopsies were performed 6 days after oocyte retrieval. The global miRNA and mRNA gene expressions in endometrial biopsies were investigated with a V4.0 miRNA probe and 22 K Human Genome Array. Fold ratios were derived to compare gene regulation between the groups. *Spp1 *and *Ang *gene expression was selected to verify the array results by RT-PCR and the protein expression of osteopontin and VEGF was determined using an immunohistochemical method.

**Results:**

There were 4 miRNA (all down-regulated) and 22 mRNA (13 up-regulated and 9 down-regulated) exhibiting differential expression between the groups on the microRNA and microarray chips. *miRNA-451, Spp1*, and *Ang *expression in RT-PCR verified the array results. Osteopontin and VEGF were also shown to have positive expression in the endometrium.

**Conclusions:**

Data from microRNA and microarray analysis suggests dissimilar endometrial receptivity in patients with high P levels on the day of hCG, and elevated osteopontin and decreased VEGF had poor pregnancy rates.

## Background

Elevated serum progesterone (P) on the day of human chorionic gonadotrophin (hCG) has been reported to occur in 20%-40% of in vitro fertilization (IVF) and embryo transfer (ET) cycles [1]. Our clinical research has shown the pregnancy outcome was much poorer (13.79 versus 44.68%) if the serum P on the day of hCG was >6.0 nmol/l. However, the potential effect of these subtle increases on implantation remains controversial. Some argue that high serum P concentrations could have an adverse effect on oocyte quality or endometrial conditions [2]. Recent clinical studies have supported this notion of decreased endometrial receptivity. Melo et al. [3] conducted experiments involving patients in oocyte donation cycles (two cycles per woman, one normal and another P elevation cycle). The recipient's pregnancy rates were similar per IVF-ET, whether or not P was elevated. Our clinical research also showed that patients with elevated P had a poor outcome of fresh ET cycles, but a similar successful outcome in frozen embryo transfer (FET) cycles compared with the normal P group [4]. These reports further confirmed the link between poor pregnancy rates and decreased endometrial receptivity in patients with elevated P, not fertilization or embryo quality.

Endometrial receptivity is a complex process, both temporally and spatially restricted. Improved endometrial receptivity and embryo preparation should lead to increased pregnancy rates and reduced early pregnancy failure [5]. It is generally assumed that the receptive window of the human menstrual cycle is limited to days 19-24 [6], and days 8-10 post-ovulation [7]. Endometrial receptivity has been extensively studied, with a number of biochemical markers for endometrial receptivity having been proposed [8], including integrin and leukaemia inhibitory factor (LIF), although none of the biomarkers has proven to be clinically useful as an indicator [9]. In fact, many factors can play roles in endometrial receptivity, making it difficult to gain a clear understanding of gene regulation in the endometrium [10]. Are there any such uterine markers of endometrial receptivity associated with higher P levels?

During the past few years, both complementary DNA (cDNA) and oligonucleotide microarray technologies have been successfully applied to the study of endometrial gene expression [11]. The availability of this technology makes it possible to investigate the endometrial receptivity process from a global genomic perspective [12]. miRNAs are small (approximately 22 nt) ribonucleotides found in both animal and plant cells which play important roles in cellular differentiation, tissue development, and apoptosis by regulating gene expression and protein translation. Therefore, the current investigation sought to identify miRNAa- and mRNAs associated with the development of endometrial receptivity by comparing global gene expression patterns in normal and elevated P endometrium. Characterization of the complex relationship between miRNAs and the target mRNAs in endometrial receptivity may assist in the determination of the molecular pathways that affect receptivity; however, that cannot be completely elucidated by mRNA gene expression analysis alone. Both miRNA array and microarray were employed to detect the main factor acting upon the poor receptivity of patients with elevated P.

## Methods

### Patient details and tissue collection

All participants, 25-36 years of age, underwent transvaginal ultrasound and physical examination; none had positive findings. The patients all had tubal or male factor infertility. Endocrinopathies, organic diseases, and other factors affecting endometrial receptivity, such as polycystic ovary syndrome (PCOS), ovarian tumor, polyps, fibroids, endometriosis, and hydrosalpinx were excluded. All patients underwent IVF-ET with gonadotrophin-releasing hormone agonist (GnRHa) and recombinant FSH (rFSH). A total of 19 endometrial samples were collected in this study and separated into 2 groups. The mean ages in normal and elevated P groups were 29.63 and 32.07 years, respectively (*P *> 0.05). Group 1 consisted of patients with normal P levels (serum P levels <4.0 nmol/L on the day of hCG) and included 7 patients who had opted out of ET in order to avoid ovarian hyperstimulation syndrome (OHSS) in IVF-ET cycles. Group 2 consisted of patients with elevated P levels (serum P levels >6.0 nmol/L on the day of hCG, which was defined by our clinical research [4]), including 12 patients who had cancelled fresh ET in hopes of improving the chances for success during frozen ET cycles. Endometrial tissue biopsies were performed by dilation and curettage 6 days after oocyte retrieval. Each endometrial sample was further divided for the following studies: one portion was used for routine histologic processing and immunohistochemistry (IHC) in paraffin blocks to assess Noyes staging; the remainder was immersed in RNA Later solution and stored at -80°C until RNA extraction. Ten samples (5 per group) were evaluated for the expression of miRNAs and mRNAs.

Informed consent was obtained for all patients undergoing endometrial biopsies; these protocols were approved by the Ethics Committee of Peking University Third Hospital.

### miRNA isolation labeling and microcrarry analysis

Five endometrial samples from patients in each group were used for microarray hybridization according to the manufacturer's instructions. Samples from all patients were detected independently. Total RNA was extracted from endometrial samples. Methylaldehyde degeneration gel electrophoresis was performed to further confirm RNA integrity. Low molecular weight RNA was isolated using the PEG solution precipitation method, according to a previous protocol [13]. The T4 RNA ligase labeling method was adopted according to the Thomson protocol [14]. Microarrays were scanned with a confocal LuxScan™scanner (CapitalBio Corp., Beijing, China); scanning settings were adjusted to obtain a visualized balanced of U6 and tRNA signals across arrays. Data were extracted from the TIFF images using LuxScan™3.0 software (CapitalBio Corp.). The microarray intensity data were analyzed by using Significance Analysis of Microarrays (SAM) software. Two class unpaired t-tests were performed to identify miRNA expressed differences among the three groups. Genes possessing a q-value equal to 0 and a fold-change >2 were considered significantly different.

### Messenger RNA profiling with microarray analysis

Messenger RNA was analyzed from samples in parallel with the miRNA extraction procedures (see Methods section above). A total of 21,522 well-substantiated human mRNA genes were analyzed using the 22 K Human Genome Array (CapitalBio Corp.). Two μg of total RNA was reverse-transcribed, and then double-stranded cDNAs (containing the T7 RNA polymerase promoter sequence) were synthesized using the CbcScript reverse transcriptase with cDNA synthesis system, according to the manufacturer's protocol (Capitalbio) with the T7 oligo (dT). To determine gene products with a significant change in levels of expression, statistical comparisons were done using the two classes unpaired t-tests in SAM software in order to identify the functional classes (gene ontology) of selected genes.

### Real-time PCR

To verify the array results, 3 samples per group were chosen. Real-time PCR reactions were performed using miRNA and mRNA specific primers for *miRNA-451 *(*miR-451*), the lowest expression of *miRNA*, *Spp1*, and *Ang *expression. The GenBank accession numbers of each gene, along with the primer sequences and PCR product sizes of the amplified fragment are listed in Table 1. Five mg of total RNA extracted from each sample was digested with RNase-free DNase I (Promega, San Luis Obispo, CA USA) and reverse-transcribed into cDNA using Superscript II reverse transcriptase (Invitrogen, Carlsbad, CA USA). mRNA of the housekeeping gene *ACTB *(forward primer: AAGTACTCCGTGTGGATCGG; and reverse primer: ACATCTGCTGGAAGGTGGAC) was used as an endogenous control. Duplicate samples of the target and reference genes were amplified in separate wells; controls without the RT step were included for each primer pair to check for any contaminants. Melting (dissociation) curves of PCR reactions were monitored to ensure a single PCR product and no primer dimmer. All experiments were repeated 40 cycles. The relative expression ratio of a target gene was calculated based upon efficiency and the CT deviation of an unknown sample versus the control (using myometrium cDNA as a calibrator); this was expressed in comparison to a reference gene [15]. The mean CT ratios were expressed as fold differences in gene expression compared with myometrium (gene expression = 1).

**Table 1:** The GenBank accession numbers of the primers and sequences and sizes of the amplified fragments used for real-time PCR analysis

Gene	**GenBank accession no**.	Sequence (5'-3')	Length	Amplicon size (bp)
*miR-451*	hsa-miR-451	RT:GTCGTATCCAGTGCAGGGTCCGAGGTATTCGCACTGGATACGACaactca AS: GCGAAACCGTTACCATTACTGA		
*Spp1*	NM_000582	R:TGGGGTCTACAACCAGCATA F:ACATCTTTGCGTTTTCTACCG	20 20	103
*Ang*	NM_001145	R:CTGTGGTTTGGCATCATAGTG F:CATGTACGTTGCTATCCAGGC	21 21	264

### Immunohistochemistry methods

After deparaffinizing sections, the section slides were rehydrated in steps with distilled water (dH2O); slides were immersed in 0.5% v/v hydrogen peroxide/methanol for 10 minutes. The antigen retrieval protocol can be carried out at this time, should this procedure be deemed necessary. Tissue sections were washed in dH_2_O twice (5 minutes each), then blocked with blocking solution (biotinylated goat anti-rabbit immunoglobulin G). Tissue sections were quickly labeled with a PAP pen before the tissue sections dried. After blocking, primary antiserum diluted in blocking solution (1:100 dilution; Santa Cruz Biotechnology, Inc., Santa Cruz, CA, USA) was added and incubated overnight at 4°C. The sections were incubated with biotinylated secondary antibody diluted in blocking reagent for 30 minutes at 25°C. Finally, ABComplex/HRP was added for 30 minutes at 25°C; sections were developed with 3, 3' diaminobenzidine tetrahydrochloride (DAB) and subsequently counterstained with haematoxylin. The primary antibody was omitted to create negative controls.

The immunostaining was scored semi-quantitatively in each section. The staining intensity and positive percentage were performed independently by two observers (Li R and Li L), and the mean value was obtained. A staining score was calculated as follows: H-score=ΣPi (i+1), where ΣPi is the sum of Pi, Pi = positive cells/100 calculated cells × 100%, and i = intensity score (negative = 0, weak = 1, moderate = 2, strong = 3). This score value could vary between 0 and 400.

### Statistical analysis

Intensity values were analyzed using the SAM method, which is a statistical technique for finding significant genes in a set of microarray experiments [16]. Two class unpaired t-tests were performed to identify mRNA differences among the three groups. Genes possessing a q-value equal to 0 and a fold-change >2 were considered significantly different. The q-value is the false discovery rate, which measures how significant the gene is. The Statistics Package for Social Sciences (SPSS) 13.0 software (SPSS Inc., Chicago, IL, USA) was used to analyze the data by Student's *t*-test and rank test. A *P *< 0.05 was considered statistically significant.

### Pathway analysis

In order to identify gene expression, the relationship between biologic context and pathways was analyzed using commercially available software (Ingenuity Systems, Redwood City, CA, USA). Ingenuity pathway analysis was performed on mRNA genes expressed in the endometrium, comparing normal and elevated P groups; this was to predict mRNA target genes of differentially expressed miRNA. A p-value < 0.05 was used to determine the statistical significance.

## Results

The relationship between miRNA and the target mRNA in endometrial receptivity was determined to assist in the determination of the molecular pathways that affect receptivity between higher and normal P levels.

### miRNA microarray screening

**Figure 1:**
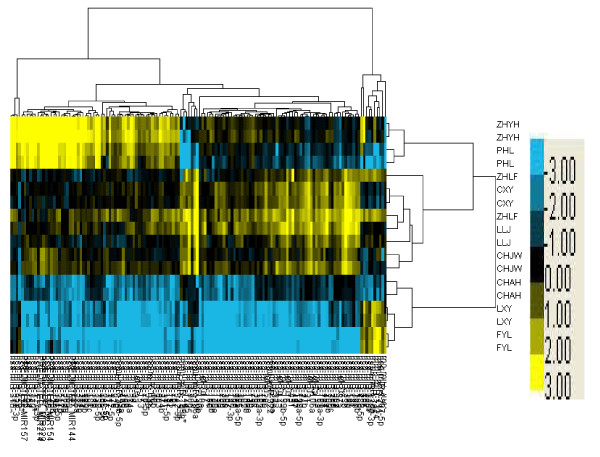
**Four down-regulated miRNAs possessing significant differential expression between normal progesterone and elevated progesterone groups (fold change <0.2)**.

A total of 4 down-regulated miRNAs demonstrated statistically significant differential expression between the control, normal P, and elevated P groups (fold change < 0.2); this implies that four miRNAs exhibited a relative decreased expression (*hsa-miR-451, hsa-miR-424, hsa-miR-125b*, and *hsa-miR-30b*; Figure 1 and Table 2).

**Table 2:** Relative expression of miRNAs decreased in endometrial samples taken during the implantation window between the groups, as shown by miRNA microarray analysis

Fold change	*q *value (%)	HUGO name
0.123	0	*hsa-miR-451*
0.135	0	*hsa-miR-424*
0.185	0	*hsa-miR-125b*
0.186	0	*hsa-miR-30b*

### mRNA microarray screening

**Figure 2:**
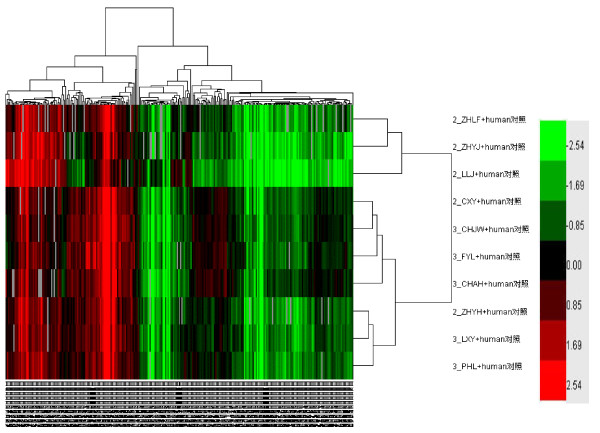
**Thirteen up-regulated and 9 down-regulated genes between normal and elevated progesterone groups (fold change >2 up-regulated and <0.5 down-regulated)**.

Total endometrial RNA from five samples per group was extracted by dilation and curettage. These RNA preparations were then used to probe the human gene microarray comprised of 21,522 genes. Only those genes with a *q *value <5% in the one class method from SAM analysis and a ratio of at least 2-fold difference among the groups, were considered. A total of 22 genes displayed significant (*P *< 0.05) decreases in expression from endometrial samples taken during the implantation window between the groups. Among the 22 mRNA genes, 13 up-regulated and 9 down-regulated were detected in the elevated P group (Figure 2 and Table 3). In an effort to further explore the biologic relationship between reduced receptivity and identified miRNA, a search of the Sanger Database for predicting mRNA targets of the *miR-451 *(the most differentially expressed, fold change = 0.12) was conducted. The Sanger Database-predicted mRNA gene targets of *miR-451 *were then cross-referenced against the 22 mRNA genes; indeed, it was shown that *miR-451 *gene targets were differentially expressed between normal and elevated P endometrium genes from the Affymetrix microarray expression analysis. Four of 22 (18%) differentially expressed genes, *Tyr *(tyrosinase precursor), *Usp16 *(ubiquitin carboxyl-terminal hydrolase 16), *Galk2 *(N-acetylgalactosamine kinase), and *Spp1 *(osteopontin precursor) were also predicted mRNA targets of *miR-451*.

**Table 3:** Relative expression of mRNAs decreased in endometrial samples taken during the implantation window between normal and elevated progesterone groups, as shown by mRNA microarray analysis

Fold change	*q *value (%)	Description	HUGO name	**GenBank accession no**.
2.7119	13.1050	Serine-protein kinase ATM	*Atm*	NM_000051,NM_138292
2.6390	5.7126	Proteinase activated receptor 1 precursor (PAR-1)	*F2r*	NM_001992
2.5389	9.0200	Osteopontin precursor (Bone sialoprotein 1)	*Spp1*	NM_000582
2.5156	5.7126	SA hypertension-associated homolog isoform 1	*Sah*	NM_202000,NM_005622
2.4192	13.1050	Lysozyme C precursor	*Lyz*	NM_000239
2.3716	9.0200	Kinetochore associated 2	*Kntc2*	NM_006101
2.1913	5.7126	Liver carboxylesterase 1 precursor	*Ces3*	NM_001266
2.1760	9.0200	N-acetylgalactosamine kinase	*Galk2*	NM_001001556,NM_002044
2.1591	13.1050	Alcohol dehydrogenase; Fe-containing alcohol dehydrogenase 1	*Adhfe1*	NM_144650
2.1208	13.1050	Heterogeneous nuclear ribonucleoproteins A2/B1	*Hnrpa2b1*	NM_002137,NM_031243
2.0778	5.7126	Influenza virus NS1A binding protein; NS1-binding protein	*Ivns1abp*	NM_016389,NM_006469
2.0611	13.1050	Lactamase, beta 2	*Lactb2*	NM_016027
2.0439	9.0200	Ubiquitin carboxyl-terminal hydrolase 16	*Usp16*	NM_006447,NM_001001992
0.4958	35.2491	Histone deacetylase 5 (HD5)	*Hdac5*	NM_005474,NM_139205
0.4796	32.6148	1-acyl-sn-glycerol-3-phosphate acyltransferase beta	*Agpat2*	NM_006412
0.4305	5.1933	Angiogenin precursor	*Ang*	NM_001145
0.4253	32.6148	LAG1 longevity assurance homolog 4	*Lass4*	NM_024552
0.4141	35.2491	Fibrinogen beta chain precursor	*Fgb*	NM_005141
0.4102	32.6148	Similar to lymphocyte antigen 6 Complex, locus G5B; G5b protein; open reading frame 31	*Np_660282*	NM_145239
0.3214	32.6148	Left-right determination factor B precursor	*Leftb*	NM_020997
0.2476	32.6148	WNT1 inducible signaling pathway protein 2 precursor (WISP-2), (Connective tissue growth factor-like protein) (CTGF-L), (Connective tissue growth factor-related protein 58)	*Wisp2*	NM_003881
0.1734	35.2491	Tyrosinase precursor	*Tyr*	NM_000372

### Real-time PCR

**Figure 3:**
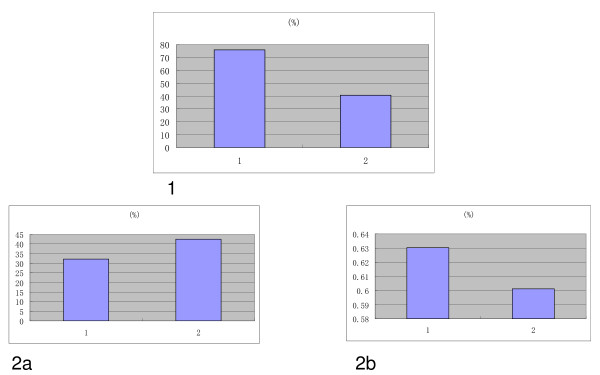
**Validation of *hsa-miR-451, Spp1, and Ang *gene expression between the groups**. Group 1: normal progesterone group; group 2: elevated progesterone group. 3 endometrium samples per group were involved. 1: *hsa-miR-451 *gene expression; 2a: *Spp1 *gene expression; 2b: *Ang *gene expression

In an effort to validate the array expression findings, one of the four differentially-expressed miRNAs (*hsa-miR-451*) was chosen for real-time PCR (RT-PCR) analysis (Figure 3); three samples were taken per group. The expressions proved to be lower in group 2 (34.3%, 47.4%, and 39.9%; average, 40.56%) than in group 1 (44.43%, 100%, and 82.85%; average, 75.76%). Two of the 22 differentially-expressed mRNA (*Spp1 *and *Ang*) were chosen for RT-PCR analysis (Figure 3). *Spp1 *was one of the 4 *miR-451 *regulated genes. We also chose 3 samples per group for RT-PCR analysis. *Spp1 *expression was higher (group 1: 12.12%, 26.65%, and 57.89%; average, 32.22% vs. group 2: 40.21%, 42.90%, and 44.17%; average, 42.43%), while *Ang *expression was the lower (group 1: 80.68%, 78.34%, and 30.05%; average, 63.03% vs. group 2, 37.36%, 87.60%, and 55.38%; average, 60.11%). These results totally supported the accuracy of the mRNA microarray analysis.

### Protein verification

**Figure 4:**
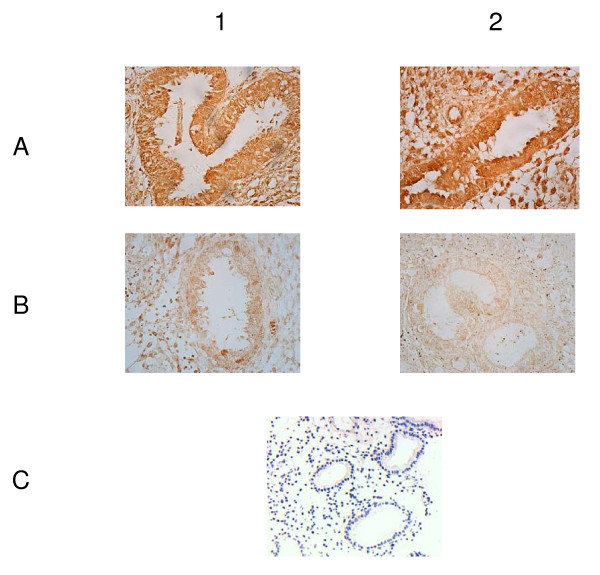
**Osteopontin and vascular endothelial growth factor (VEGF) proteins expressed in normal, and elevated progesterone endometrium by immunohistochemistry methods**. Group 1: normal progesterone group; group 2: elevated progesterone group. A: Osteopontin; B: vascular endothelial growth factor; C: negative control.

In order to explore the biological significance of the *Spp1 *and *Ang *genes, their expression in normal and elevated P endometrium were analyzed by imunohistochemistry methods. The results demonstrate that osteopontin and vascular endothelial growth factor (VEGF) proteins were found in both normal and elevated P endometrial tissues, both stromal and glandular cells. The osteopontin was staining more intense and VEGF less intense in women with high versus normal serum P group, as calculated by the H-score (Figure 4 and Table 4).

**Table 4:** Different expression of OPN and VEGF in endometrial samples taken during the implantation window between normal and elevated progesterone groups, as shown by imunohistochemistry analysis

	Group 1	Group 2	*t*	*P*
Samples	7	12		
OPN	313 ± 29	362 ± 19	-4.036	0.003
VEGF	343 ± 34	304 ± 28	2.683	0.016

## Discussion

Successful implantation of embryos is an important physiologic event in the establishment of pregnancy [17]. High serum P concentrations could have an adverse effect on endometrial conditions, a pattern observed through our clinical research [4]. However, what factors affect endometrial receptivity of high P patients? Global gene expression analysis has been used to search the etiologic factors of many diseases [18]. In recent years, many studies have been conducted to explore the cause of endometrial receptivity reduction [19]. Our study used a gene array to investigate the expression of differential genes in the endometrium in patients with elevated P; this was chosen because of the extremely poor outcome in IVF-ET treatment observed in these individuals. Some of the 22 genes identified proved to be involved in invasive growth and the cystoskeleton; among them, a number have been previously reported to affect endometrial receptivity directly or indirectly, including oesteopontin [20] and angiogenin (which regulates VEGF expression) [21].

We have utilized genome-wide analysis to identify a series of miRNA which may contribute to reductions in endometrial receptivity. By executing a parallel analysis of mRNA levels in a subset of these samples, we have identified a panel of mRNAs that may influence endometrial receptivity. A proportion of these mRNA genes have been previously reported as predicted targets of the miRNA [22]. Using the Sanger miRNA database, several of the differentially-expressed genes have been identified as target genes for miRNA in this study, which may play an important role in reducing endometrial receptivity. Furthermore, we have identified 4 miRNAs and 22 mRNA genes that were differentially-expressed between normal and elevated P endometrium. Using the Sanger database, 4 of the 22 mRNA genes were identified as predicted targets of the *miRNA-451*; several of these mRNA genes are associated with endometrial receptivity.

The relationship between miRNA gene expression and the mRNA targets is very complicated. It is believed that miRNA influences cell function via post-transcriptional modulation [23]; thus, the effect of miRNA is largely directed toward post-transcriptional activity. The post-transcriptional down-stream effects of miRNA on translation may operate on a feedback-loop, with consequences impacting mRNA transcription of the miRNA predicted targets. Moreover, the effect of miRNA on messenger RNA degradation may also result in changes in mRNA levels that may be detected through an array expression analysis.

Osteopontin is an important factor because of its involvement in the implantation process. This glycoprotein is a ligand for α v β3 integrin; osteopontin mediates cellular adhesion and migration during embryo implantation and is regulated by P. Its maximal expression in endometrial epithelial cells has been observed during the window of implantation [24]. VEGF and its receptor had intense expression in the endometrial epithelial cells of the implantation widow and also showed stromal immunoreactivities by the time that secondary villi differentiate into tertiary villi [25]. When there were some abnormal expressions of cytokines, such as OPN and VEGF, the endometrial receptivity decreased, which led to poor pregnancy rate in IVF patients. A better pregnant outcome in frozen ET cycles may be achieved when the patient has an elevated P following the administration of hCG.

## Conclusions

We performed a gene pathway analysis on miRNA and mRNAs in order to report the effects of this differential expression between normal and elevated P. This pathway analysis identified osteopontin and angiogenin to play an important role in endometrial receptivity of elevated P. All this data may provide a reason to explain the reduced pregnancy rate in patients with elevated P, thus creating new markers for improved treatment for patients undergoing IVF-ET with elevated P levels.

## Competing interests

The authors declare that they have no competing interests.

## Authors' contributions

RL carried out the operation, participated in the sequence data collection and analysis and drafted the manuscript. JQ instigated the study, and participated in its design and coordination and helped to draft the manuscript. Both authors read and approved the final draft.
